# Phenotypic Characterization of a Genetically Diverse Panel of Mice for Behavioral Despair and Anxiety

**DOI:** 10.1371/journal.pone.0014458

**Published:** 2010-12-29

**Authors:** Brooke H. Miller, Laura E. Schultz, Anisha Gulati, Andrew I. Su, Mathew T. Pletcher

**Affiliations:** 1 Department of Neuroscience, The Scripps Research Institute-Scripps Florida, Jupiter, Florida, United States of America; 2 Department of Molecular Therapeutics, The Scripps Research Institute-Scripps Florida, Jupiter, Florida, United States of America; 3 Genomic Institute of the Novartis Research Foundation, San Diego, California, United States of America; RIKEN Brain Science Institution, Japan

## Abstract

**Background:**

Animal models of human behavioral endophenotypes, such as the Tail Suspension Test (TST) and the Open Field assay (OF), have proven to be essential tools in revealing the genetics and mechanisms of psychiatric diseases. As in the human disorders they model, the measurements generated in these behavioral assays are significantly impacted by the genetic background of the animals tested. In order to better understand the strain-dependent phenotypic variability endemic to this type of work, and better inform future studies that rely on the data generated by these models, we phenotyped 33 inbred mouse strains for immobility in the TST, a mouse model of behavioral despair, and for activity in the OF, a model of general anxiety and locomotor activity.

**Results:**

We identified significant strain-dependent differences in TST immobility, and in thigmotaxis and distance traveled in the OF. These results were replicable over multiple testing sessions and exhibited high heritability. We exploited the heritability of these behavioral traits by using *in silico* haplotype-based association mapping to identify candidate genes for regulating TST behavior. Two significant loci (-logp >7.0, gFWER adjusted p value <0.05) of approximately 300 kb each on MMU9 and MMU10 were identified. The MMU10 locus is syntenic to a major human depressive disorder QTL on human chromosome 12 and contains several genes that are expressed in brain regions associated with behavioral despair.

**Conclusions:**

We report the results of phenotyping a large panel of inbred mouse strains for depression and anxiety-associated behaviors. These results show significant, heritable strain-specific differences in behavior, and should prove to be a valuable resource for the behavioral and genetics communities. Additionally, we used haplotype mapping to identify several loci that may contain genes that regulate behavioral despair.

## Introduction

Major affective disorders, including depression, anxiety, bipolar disorder, and schizophrenia, have a combined lifetime prevalence rate of approximately 25% [1]. Psychiatric disorders are also a risk factor for a host of disorders ranging from substance abuse to heart disease [2]. Although most forms of psychiatric disease exhibit 40–60% heritability, very few causative genes have been conclusively identified [3], [4], [5]. Several major hurdles have impeded the success of genome-wide association scans, including genetic heterogeneity, epistatic gene interactions, and the role that the environment plays in the development and expression of the disease [6].

The use of model organisms can reduce the impact of confounding factors on complex phenotypes. In humans, depression is characterized by a combination of cognitive, emotional, and physiological symptoms; because it is difficult to model many of these symptoms in animals, tests generally focus on a single behavior that represents a specific human endophenotype. Two of these tests, the Tail Suspension Test (TST) and Forced Swim Test (FST), measure stress-induced coping mechanisms [7], [8]. In both the TST and FST, an animal is faced with an inescapable stress (being suspended by the tail or trapped in a beaker of water), and the immobility that eventually develops is believed to represent a state of behavioral despair. The validity of these tests as a model for depression is suggested by a number of factors. First, clinically effective human treatments such as antidepressants reduce TST and FST immobility [9], [10]; second, manipulation of genes known to be involved in depression in humans affects TST and FST performance [11], [12], [13], [14]; and, finally, mice bred to express a behavioral or physiological “depressive” phenotype show increased immobility [15], [16], [17]. Similarly, the Open Field task is considered a valid model of anxiety, as it puts an animal in a stressful situation (an open field) and takes advantage of an ethologically relevant response (thigmotaxis) to measure general anxiety [18].

It has previously been observed that inbred mice show strain-dependent responses to behavioral tasks, indicating that factors that regulate performance in these tasks are under genetic control [19]. The common inbred strains have been shown to encompass phenotypic variation on par with human populations for a number of complex behavioral and metabolic traits [20], [21]. Notably, a number of inbred mouse strain differences have been documented for physiological and behavioral phenotypes relevant to human mood disorders. For example, serotonin levels and serotonin receptor binding are higher in C57BL/6J mice than in BALB/cJ [22], while BALB/cJ mice have higher baseline and stress-induced corticosterone levels than several other strains [23]. Researchers have also identified strain differences in hippocampal size [24], serotonin receptor distribution in the brain [25], whole brain monoamine and catecholamine content [26], [27], [28], and hippocampal neurogenesis [29], [30].

Phenotyping of inbred mouse strains offers multiple benefits [19]. First, because the genetics of these populations are fixed, each strain only has to be phenotyped once, and the results can be interrogated repeatedly without requiring additional animals. Second, knowledge of specific strain characteristics (high versus low anxiety, drug metabolism, etc.) allows researchers to select the most appropriate strain for their research. The public availability of phenotype data in the Jackson Lab's Mouse Phenotype Database (http://phenome.jax.org) has been a significant resource for the community [31]. Finally, the mosaic genetic structure and phenotypic diversity of inbred strains can be leveraged to identify biologically important genetic loci for complex traits using haplotype association mapping (HAM) [32], [33]. This methodology has been successfully used to identify genes that play a role in acetaminophen-induced liver injury [34], regulation of oxidative phosphorylation in vivo [35], expression of a family of genes involved in drug detoxification [36], anxiety [37], bone mineral density [38], lung tumor susceptibility [39], and drug metabolism [40]. In each of these cases, work was done to validate that the genes underlying the significant association peaks directly regulate the end phenotype. In the case of the acetaminophen-induced toxicity, the predisposing loci were shown to directly translate to the human population, meaning that a statistically significant portion of the susceptible population carried a particular allele of the same gene implicated in the mouse haplotype association mapping. While most of these studies were successful in using a modest number of inbred strains to power their associations, it has been estimated that a larger set will be required to identify genes that have smaller effect sizes and reduce false positives [41]. Behavioral traits in particular are expected to be regulated by a large number of causative genes that each exert a small influence [6].

In the present study, we selected 33 inbred laboratory mouse strains, including 31 classical inbred strains and 2 wild-derived strains, based on the Mouse Phenome Database priority list and the availability of single nucleotide polymorphism (SNP) data. We phenotyped almost 800 mice for 3 behavioral traits: behavioral despair (immobility in the TST), general anxiety (thigmotaxis in the Open Field), and general motor activity (distance traveled in the Open Field). In each of these tests, there were clear strain-specific differences, and we were able to draw correlations between the degree to which performance on each task influenced performance on the other behavioral tasks. Finally, we used strain-specific performance in the TST as a quantitative phenotype for haplotype mapping and identified two ∼300 kb genetic loci with a lod score >7 (corrected p value <0.04), indicating that inbred strain phenotyping and haplotype mapping can successfully be applied to identify risk loci for complex phenotypes.

## Materials and Methods

### Animals

Male mice from 33 inbred strains (Table S1) were obtained from The Jackson Laboratory (Bar Harbor, ME) at 3–5 weeks of age. Mice were housed at a density of 3 per cage and maintained on a 12∶12 light:dark cycle (lights on  = 0700 h). Food and water were available *ad libitum*. Mice were allowed to acclimate for 1 month prior to behavioral testing. All animal procedures were approved by the Scripps Florida Institutional Animal Care and Use Committee under protocol 06-019.

### Behavioral testing

Behavioral testing took place between 1300 h and 1600 h in a room separate from the colony room. Mice were brought into the behavioral testing room immediately prior to testing and removed immediately afterward, and the behavioral apparatus was cleaned with a disinfectant between each mouse. Four to eight mice were tested at a time, and strains were randomized across day, time of testing, and equipment. Each individual was only tested once in each task.

Tail Suspension was performed using a Mouse Tail Suspension setup (Med Associates, Georgia, VT). In this test, the mouse is placed inside a 3-sided cubicle and suspended by its tail from a hanger attached to a precision linear load cell that measures activity. Measurements were taken in 200ms increments for 7 minutes, with threshold  = 3 and gain  = 8. Because all mice were uniformly active for the first minute, percent immobility was calculated by determining the time spent immobile during the last 6 minutes of the test. Mice that climbed their tail or fell off the hanger were excluded from analysis. A total of 780 mice were successfully phenotyped in the TST.

One week later, mice were tested in a Med Associates mouse Open Field contained within an environmental chamber that provided white noise and low, indirect lighting. These boxes measure activity using infrared beam breaks on the x, y, and z axes. Mice were placed in the center of the field and allowed to explore freely for 10 minutes. Center time was calculated as the percent of time spent in the center 25% of the field, and distance traveled was measured in total cm covered. A total of 797 mice were phenotyped in the Open Field. Statistics were performed using Pearson's r test for correlation (Microsoft Excel). A p-value test for was conducted for each of the correlation coefficients to determine the statistical significance of the Pearson's value. A one-way ANOVA (JMP, SAS Statistics) was used to calculate behavioral phenotype differences between haplotypes.

All behavioral data are publicly available under the record “Pletcher1” in the Mouse Phenome Database (http://phenome.jax.org).

### Corticosterone Radioimmunoassay

Trunk blood was collected following live decapitation. All collections were performed between 1100 h and 1500 h, and blood was collected within 30 seconds of removing the mice from the colony room. Blood was allowed to clot on ice, and then serum was separated out and stored at -80°C. Corticosterone was measured from 10 µl of serum using a double antibody RIA kit (Diagnostics Products Corp. Los Angeles, CA). The minimum detectable level was 0.5 µg/dl, and interassay/intrassay coefficients of variance were 12.48% and 6.11%, respectively. The RIA was performed by the Ligand Assay Core at Northwestern University.

### Heritability calculation

For each phenotype, heritability was calculated using 8–16 randomly selected scores from each inbred strain. The number of scores used for calculating heritability was fixed at the lower level (8) by the maximum number of scores available for some strains, and at the upper level (16) by the maximum allowed by the heritability algorithm. Phenotype values were fit using an ANOVA model with a single fixed effect (strain), and heritability was calculated as the adjusted percentage of variance explained. In order to ensure that estimates were not affected by outliers, heritability was calculated twice per phenotype using separate individuals each time. For heritability calculations based on data downloaded from the Mouse Phenome Database, up to 16 scores per strain from male mice only were used for each phenotype, and only the strains used in the present experiment were included in heritability calculations.

### Haplotype mapping

*In silico* haplotype association mapping was used to link TST performance to genomic regions with the publicly available SNPster web tool (http://snpster.gnf.org), which correlates strain-specific log-transformed phenotype data with a high density SNP map (roughly 140,000 SNPs or 1 SNP/6 kb) [32], [42]. The SNPster algorithm infers haplotype by use of a sliding 3-SNP window and calculates an ANOVA F-statistic for each potential haplotype x phenotype association. The F-statistic is weighted to reduce the importance of association scores that are largely driven by closely related strains, such as multiple lines from the C57-related strains [41]. A generalized family-wise error rate (gFWER) model is used to set a significance level adjusted for multiple testing. Standard testing conditions (weight estimate  = 3, –logP threshold  = 2.5, SNP window size  = 3) were used. For a locus to be included, it had to have at least 2 haplotypes with at least 5 members each. 1000 genome scans were performed and an F-test was used to compute significance. gFWER calculations (1000 permutations, *k* = 10) set a raw –logP value of 7 or higher as significant (adjusted p value <0.05), and a raw –logP value of 5.65 or higher as suggestive (adjusted p value <0.1).

### SNP genotyping and re-sequencing

SNP genotyping was performed on genomic DNA from the 33 phenotyped inbred mouse strains using a single-base extension reaction on the Sequenom genotyping platform. In this two-step process, the region containing the SNP is amplified by PCR, and then a primer ending at the polymorphic site is used for the single-base extension reaction. Completed genotyping reactions were spotted in nanoliter volumes onto a matrix arrayed into 384 elements on a silicon chip (Sequenom SpectroCHIP), and the allele-specific mass of the extension products was determined by matrix-assisted laser desorption ionization, time-of-flight mass spectrometry (MALDI-TOF MS). SPECTROTYPER software was used for data analysis.

Re-sequencing primers (IDT, Coralville, IA) were designed to include all translated exon regions for the target genes *Socs2*, *Nudt4*, and *Tmem108*. Products were amplified from reference genomic DNA for AKR/J, C57BLKS/J, BTBR T+ *tf*/J, CBA/J, DBA/2J, and FVB/NJ, prepared for sequencing using a pre-sequencing 96-well kit (Millipore, Billerica, MA), and sequenced using a Big Dye reaction (Applied Biosystems, Foster City, CA) by the University of Chicago DNA Sequencing Core. Sequence traces were aligned with and compared to reference sequences from NCBI in order to identify strain-dependent nucleotide differences.

## Results

### Behavioral phenotyping

**Figure 1:**
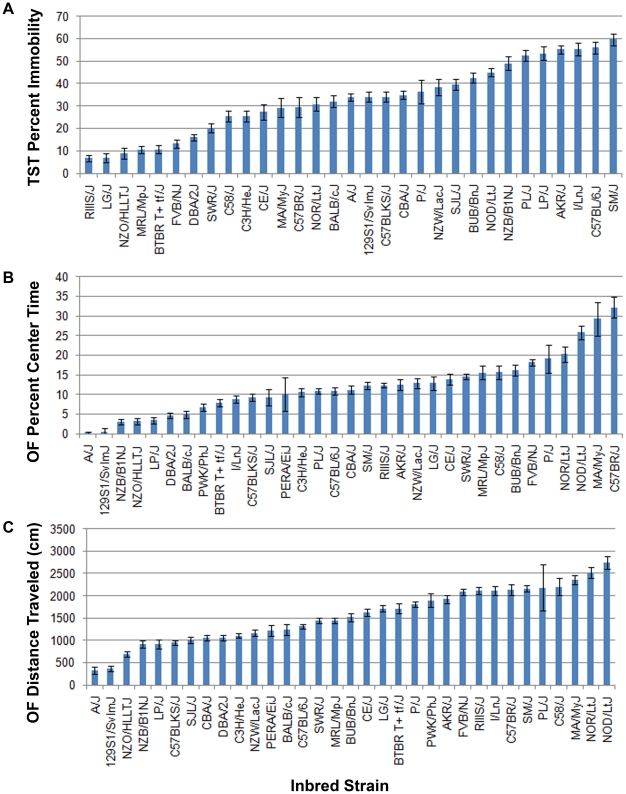
33 inbred strains were phenotyped for behavioral despair (*A*, percent immobility in the TST), anxiety (*B*, thigmotaxis in the Open Field), and locomotor activity (*C*, distance traveled in the Open Field). Strain-specific differences were observed for all phenotypes. Error bars indicate SEM.

We used the Tail Suspension Test to measure behavioral despair. To reduce intra-strain variability, only males were used, and all mice were tested at 7–10 weeks of age. Each strain was represented by a minimum of 8 and a maximum of 49 individuals, with an average group size of 25 individuals. In total, 890 mice were phenotyped, although 110 mice were excluded due to tail-climbing. As described by other groups using smaller strain sets, we observed a large, strain-dependent distribution in the duration of immobility [19] (Figure 1A, Table S2). The time spent immobile across the final 6 minutes of the 7-minute test ranged from 6.7% (RIIIS/J) to 59.6% (SM/J). Standard deviation ranged from 7–17% (average  = 11%). As noted by previous groups, several strains had a propensity for tail-climbing during the test: approximately 35% of C57BL/6J mice, 18% of DBA mice, and all mice from the two wild-derived strains climbed their tails, but no tail-climbing was observed in other strains [43]. Mice that climbed their tails were excluded from analysis; all subsequent analysis was performed using the 780 successful measurements.

In the Open Field, as in the TST, there was a clear effect of strain on both the percent of time spent in the center 25% of the field (Figure 1B) and distance traveled (Figure 1C, Table S2). All strains preferred the periphery to the center of the field, but 129S1/SvImJ and A/J were strongly thigmotaxic, while C57BR/J, MA/MyJ, and NOD/LtJ were much less so. Within-strain SD was generally lower for % center time than for TST immobility, averaging only 6% (range 1–16%); however, the overall % center time range was also lower (0.5–32%).

We observed a strongly significant correlation between distance traveled and % center time (r = 0.75, p<0.0001). In contrast, there was no correlation between TST immobility and OF thigmotaxis (r = −0.06, p = .75) or between TST immobility and OF distance traveled (r = 0.05, p = 0.79). There was also no correlation between baseline corticosterone and TST immobility (r = 0.03, p = 0.87), OF center time (−0.10, p = 0.59), or OF distance traveled (−0.11, p = 0.56).

### Heritability

In order to determine the relative contribution of genetics and environment to the present results, we calculated heritability using an ANOVA model. For each of the 3 behavioral measures, data from 14 mice per strain were used. The genetic component was found to account for an average of 64% of TST immobility across all strains, 62% of OF distance traveled, and 57% of OF % center time. By comparison, body weight, which we also measured for each strain, was 86% heritable.

### Haplotype mapping and candidate gene identification

**Figure 2:**
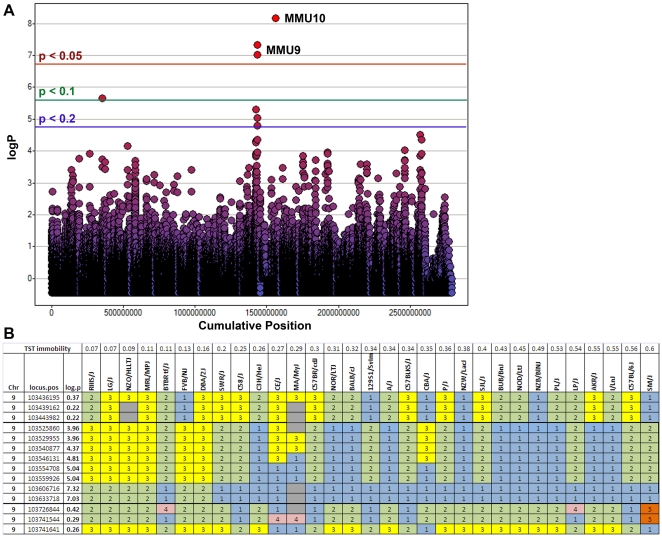
Haplotype association mapping was used to identify SNPs that associated with TST phenotype. (*A*) Three SNPs, one on MMU10 and 2 adjacent SNPs on MMU9, exceeded the significance threshold (gFWER-adjusted p<0.05). Individual SNP logP values are plotted against cumulative genomic position (bp). P value thresholds are derived from gFWER calculations. (*B*) Haplotype block pattern at the significant MMU9 locus. Strains are plotted along the x-axis from smallest to largest TST percent immobility; haplotype group is indicated by color/number. All significant and suggestive loci consisted of a core set of ∼5 unrelated, low-immobility strains.

**Figure 3:**
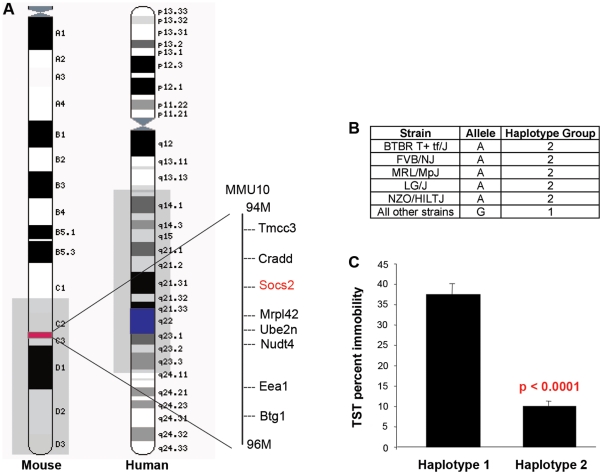
Mouse: human synteny at the MMU10 locus. (*A*) Ideogram maps of MMU10 and HSA12. The MMU10 locus (in red) is syntenic to a region on HSA12 (blue) that has been linked to both major depression and bipolar disorder. The brain-expressed gene *Socs2* lies within this locus in both mice and humans. (*B*) Resequencing identified an A/G polymorphism in the putative promoter region upstream to the *Socs2* transcriptional start site. The “A” allele is carried by the low TST immobility haplotype group, while the “G” allele is found in the haplotype group consisting of higher immobility strains. (*C)* The difference in average TST values between the “A” and “G” haplotypes is highly significant (p = 5.97^−10^).

We used haplotype association mapping to identify two genomic regions that had a gFWER-adjusted p value <0.05, and an additional region with an adjusted p-value  = 0.1 (Table 1, Figure 2). The first significant region was a 290 kb locus on MMU10 (-logp  = 8.17, gFWER-adjusted p value  = 0.033) that contained the genes *Mrpl2*, *Ube2n*, and *Nudt4*, and was immediately adjacent to *Socs2*. The second significant locus spanned a 300 kb section of MMU9 (-logP  = 7.32, gFWER-adjusted p value  = 0.041) and only contained the gene *Tmem108*. This analysis also identified several candidate regions that did not meet the stringent gFWER cutoff but still generated suggestive lod scores with adjusted p values <0.2. In order to identify candidate genes, we genotyped all known SNPs and resequenced the coding regions of the brain-expressed candidate quantitative genes *Tmem108* (NM_178638.3), *Socs2* (NM_007706.3), and *Nudt4* (NM_027722), located in or near the two significant TST loci. SNP genotyping identified two SNPs, one immediately upstream of the *Socs2* start codon, and a second located within the *Socs2* exon 2 UTR, that exhibited the same strain distribution of alleles (A/G) as the haplotype pattern driving the significant SNPster association on MMU10 (Figure 3). No relevant changes were observed in the coding regions of any of the genes.

**Table 1:** Significant or suggestive TST immobility QTLs identified by haplotype mapping.

Chrom	Mb	-logP	p value	rsID	Candidate Genes
MMU10	94.88–95.17	8.174	0.033	rs6196828	*Socs2, Mrpl42, Ube2n, Nudt4*
MMU9	103.44–103.74	7.324	0.035	rs29941513	*Tmem108*
MMU2	145.75–146.97	5.658	0.095	rs3696377	*Gm144, Xrn2*
MMU9	92.18–92.39	5.302	0.143	rs33747790	*Plscr2, Plscr4*

Haplotype mapping of the OF phenotypes returned no significant loci, although other groups have had success using inbred mouse strain haplotype mapping to identify loci associated with locomotor activity and anxiety [44], [45].

## Discussion

In the present experiments, we phenotyped 33 inbred mouse strains for immobility in the Tail Suspension Test and anxiety and activity in the Open Field. We found that these behavioral measures were consistently highly heritable, strain-specific, and had a broad phenotypic range similar to what might be expected in a human population. Our results suggest that performance in these behavioral tasks should be amenable to genetic dissection.

### Behavioral Despair

Although behavioral despair models only one aspect of human depression, it has both high face validity, as genes that are known to be involved in human depression affect mouse TST performance, and high predictive validity for antidepressant efficacy [8]. Therefore, the identification of genes involved in the regulation of TST immobility represents a forward genetic approach to identifying genes that may play a role in human depression.

We found a very strong effect of strain background on TST immobility. While other groups have examined TST performance in up to 12 strains at one time [46], [47], [48], a number of the strains we tested have not previously been phenotyped for behavioral despair. Furthermore, environmental factors (handling, age, sex, previous behavioral testing) and methods of measurement (manual versus automated, video tracking versus load displacement) have been shown to affect performance in behavioral tasks [48], [49]. Therefore, we expected some deviation between our raw immobility scores and previously published reports. While this was indeed the case, we found that the general strain rank that we observed is in agreement with results from other labs that have compared multiple inbred strains. For example, C57BL/6J are among the most immobile of strains (56% immobility), while A/J and 129/SvImJ are in the middle of the range (34% and 31% immobility, respectively) and DBA/2J and BTBR T+ tf/J represent some of the least immobile strains (16% and 11% immobility, respectively) [50], [51]. We compared our strain immobility scores with those of Trullas and colleagues, who evaluated C57BL/6J, DBA/2J, A/J, BALB/cJ, C3H/HeJ, NZB/B1NJ, and CBA/J [50], and Yoshikawa and colleagues, who tested DBA/2, C3H/He, BALB/c, and C57BL/6 [52], and found that our immobility scores had a Pearson's *r* correlation value of .67 (p = 0.05) and .65 (p = 0.18), respectively. The correlation between our TST values and those of Liu and colleagues was lower (*r* = .50, p = 0.17); however, that group observed a range of only 63–70% immobility among 6 strains (A/J, SWR/J, FVB/J, BALB/cJ, LP/J, C3H/HeJ), while in our hands these same strains ranged from 13% (FVB/NJ) to 54% (LP/J) immobility [47]. It should be noted that no other multi-strain surveys of TST behavior have produced immobility values similar to the Liu et. al results.

Reverse genetic studies using knockout or knockdown mouse models have identified a number of genes, primarily genes involved in monoaminergic neurotransmission and the stress response axis, that affect TST immobility [8], [53]. Other studies have identified non-synonymous coding SNPs in serotonin pathway genes, including a SNP in the serotonin transporter *Slc6a4* that regulates serotonin reuptake, and a SNP in *Tph2*, the rate-limiting enzyme in brain serotonin synthesis, that affect antidepressant response [54]. It is possible that a SNP variant in one of these genes is responsible for some of the strain-specific TST phenotypes that we observed. To address this possibility, we looked for an association between SNP variants and TST immobility for known coding SNPs in *Tph2*, *Slc6a4*, all serotonin receptors, and the genes for corticotrophin releasing hormone (*Crh*) and the *Crh* receptors. The C57BL/6J variant of the *Slc6a4* SNP, which reduces serotonin transport, is found in 2 of the strains we phenotyped, C57BL/6J and C57BR/J. These strains have fairly different immobility scores (56% and 30%, respectively), so this SNP is unlikely to affect baseline immobility. Similarly, A/J, BALB/cJ, and DBA/2J possess the same *Tph2* exon 11 SNP variant but have very disparate immobility scores, suggesting that the *Tph2* SNP does not have a strong effect on TST immobility. Several serotonin receptors and *Crhr2* also contain non-synonymous coding SNPs, but none associate with the strain-dependent TST immobility scores we observed. Therefore, while complete deletion of serotonin pathway genes has a clear effect on behavioral despair, SNP variations within these genes do not appear to drive the propensity for TST immobility in common inbred strains.

### Open Field behaviors

Overall immobility in the TST is likely to be the sum of a number of separate behavioral and physiological processes that include a propensity for learned helplessness, defensive behaviors, general activity levels, the stress response, and sensory or motor impairments [8], [55]. All of these measures have been shown to vary between inbred strains. For example, BALB shows a greater behavioral and physiological response to acute restraint stress than C57BL/6, and DBA and BALB have higher baseline corticosterone levels than C57BL/6 [23], [56]. Additionally, groups such as Mhyre and colleagues have found significant differences in total activity and brain neurochemistry among 15 inbred strains [57].

In order to determine the role that the stress response and general activity levels might play in the expression of TST behavior, we phenotyped all 33 strains in the Open Field test. In the OF, overall distance traveled during the test is used as a measure of general activity, and thigmotaxis (preference for the wall versus the center) is used as a measure of anxiety that is responsive to benzodiazepine treatment [58]. There was a clear effect of strain on both distance traveled and the percent of time spent in the center 25% of the field. All strains preferred the periphery to the center of the field, but certain strains did so much more strongly. As with the TST, raw strain % center time values differed, but strain rank was consistent with previously published studies. Bothe and colleagues evaluated 14 inbred strains in the OFT and found the 129 and A/J strains to be least active and most thigmotaxic, and NOD to be most active [59]; Bolivar and colleagues observed a similar aversion for the center in 129 and A/J strains [60]. Using a different measure of anxiety, the elevated plus maze, Wahlsten and colleagues found that A/J was much less active than four other inbred strains [61]. These results are consistent with our data.

We compared our OF and TST scores to determine whether there was overlap in the phenotypes being evaluated. There was no significant correlation between TST immobility and either center preference or distance traveled in the OF. We also found no correlation of baseline corticosterone levels between performance in either the TST or OF. These results suggest that, under baseline conditions, the genetic factors underlying TST performance are likely to be somewhat distinct from those regulating motor activity, general anxiety, and the stress response. This does not, however, invalidate the many reports that have identified an association between stress and depression in humans, or between chronic stress and behavioral despair in animal models [16], [62]. It is important to note that the behaviors reported here were measured in animals that were not genetically, behaviorally, or chemically manipulated to have an increased propensity for stress or anxiety, which may affect associations between the two phenotypes.

### Heritability

The ability to accurately associate behavioral phenotypes with genotype depends on the degree to which the behavior is genetically determined. Although the ANOVA calculation used here measures heritability in a broad, rather than gene-specific, manner, the results suggest that TST and OF behaviors have a strong genetic component. We used the same genetic component calculation to determine the heritability of several inbred strain phenotypes available in the Mouse Phenome Database. Some physiological measures, including HDL cholesterol level [63], red blood cell number [64], and daily food intake [65], were under stronger genetic regulation than our behavioral measures, but a surprising number were not, including blood glucose [66] and percent body fat [67]. The heritability observed in our TST and OF data was often significantly higher than that of other behavioral measures in the MPD. For example, pre-pulse inhibition [68] and daily activity [65] were only 37% and 51% heritable, respectively. This analysis suggests that environment has a minimal effect on the variance observed, meaning that the phenotypes observed in the TST and OF should be highly amenable to genetic dissection.

A number of researchers have noted the effect of environment and handling on behavioral performance [61], [69]. One group found that environmental factors accounted for significantly more variance in a nociceptive response than genetic factors (42% and 27%, respectively) [69]. Another group measured multiple behaviors, including performance in the open field, plus maze, and water maze, in 8 inbred strains in 3 separate labs; despite careful standardization of housing, care, and testing procedures, the testing environment was often more influential than genetic background [49]. These results have clear implications for any experiment that seeks to use behavioral differences among strains as a phenotype for QTL analysis. We attempted to minimize environmental factors by standardizing housing density, handlers and handling procedures, and post-shipment recovery time. We phenotyped mice from the same strains multiple times over the course of almost two years and found minimal differences in behavior: for example, the first group of DBA/2J mice phenotyped had an average immobility of 13%, while the most recently phenotyped DBA/2J mice, tested up to 19 months after the first set, averaged 14.8% immobility. These data suggest that our procedures were effective at reducing within-lab variability. Additionally, the significant correlation of rank placement of strains in the behavioral tests in our lab with previously published results indicates that the behaviors we observed are fairly resistant to environmental effects [8], [59].

### Haplotype mapping and candidate gene identification

Genome-wide analyses of the genetic polymorphisms underlying complex behavioral traits have not historically been successful at identifying causative genes. In humans, family linkage studies have low statistical power and can usually only identify Mendelian traits; in mice, QTL studies using F2 or recombinant inbred (RI) crosses have poor genetic resolution, with an average locus size greater than 20 Mb [70]. Five groups have used traditional QTL analysis to identify loci involved in the regulation of behavioral despair in mice. Yoshikawa and colleagues used an F2 intercross of the C57BL/6 and C3H/He strains to identify TST- and FST-associated loci on MMU4 and MMU11 [52]. Liu and colleagues, using an F2 cross of the NMRI and 129S6 strains, identified 3 significant baseline TST loci on MMU5, MMU12, and MMU18 [71]. More recently, Lad and colleagues used 24 BXD recombinant inbred strains to identify candidate loci on MMU4 and MMU15 [48]. TST performance has also been measured in the DeFries High and Low mice, lines selectively bred for high or low anxiety from an F2 cross of BALB/c and C57BL/6. This mapping effort identified four significant loci on MMU3, MMU5, MMU11, and MMU19 [72]. Notably, of almost twenty TST-associated QTLs that have previously been identified, only one, on MMU4, has been replicated in a second study, and only two groups have validated candidate genes [73],[74].

Alternatively, haplotype association mapping has the potential to identify novel quantitative trait genes and pathways with high genetic resolution. Although this approach has been successfully utilized to identify biologically relevant genes that underlie complex, multigenic phenotypes, there are concerns associated with the technique. Primarily, it has been suggested that a limited strain set combined with redundant population substructures across the genome will result in an unreasonably high false positive rate [75], [76]. Therefore, significant follow-up validation is required of findings resulting from HAM analysis in order to provide the absolute proof of the validity of the genotype/phenotype relationship. However, the multiple published validations of the HAM methodology indicate there is value in running the computationally cheap and relatively quick analysis as means to gain potential insights into the behavior genetics at play in this combination of inbred strains.

In the present experiment, we identified two loci of ∼300 kb each with a lod score >7 and a genome-wide FWER-adjusted p value <0.05. Several other loci with suggestive p values (lod >5.0, p<0.20) were also identified (Figure 2A). Each of these regions contains a limited number of brain-expressed genes that may regulate performance in the TST. For all regions, the most significant haplotype x phenotype associations were driven by an inferred haplotype pattern that always included the low-immobility strains LG/J and MRL/MpJ and also included at least two or more of five other low-immobility strains (NZO/H1LtJ, RIIIS/J, BTBR tf/J, FVB/NJ, and DBA/2J), while typically excluding every other strain (Figure 2B). Genealogical trees created from microsatellite and SNP data indicate that these 7 strains represent at least 5 of the separate ancestral lineages described by Beck and colleagues [77]. Such divergent ancestry suggests that the association of these strains is not spurious, and instead represents true phenotype/genotype associations.

The significant locus on MMU10 is syntenic to a region on human chromosome (HSA) 12 (Figure 3A). HSA12q23, is one of a very few loci to be associated with major depressive disorder (MDD) and bipolar disorder (BPD) in multiple experimental cohorts, including 497 European and American sibling pairs (recurrent depression) [78], 1,890 individuals from 110 Mormon pedigrees (major depression) [79], and a small European pedigree affected by both Darier's disease and bipolar disorder [80]. Only two candidates near or within this locus are expressed in the brain: *Socs2*, which is involved in neurogenesis, and *Nudt4/Dipp2,* which regulates phosphatase signaling in the brain. In order to indentify candidate quantitative phenotype genes, we resequenced the *Socs2* and *Nudt4* coding regions and genotyped SNPs within the MMU10 locus (Figure 3B). Although we did not identify any gene expression or coding changes that co-segregated with TST performance, we found 2 SNPs near or within *Socs2* that, unlike all surrounding SNPs, exhibited the same strain distribution of alleles (A/G) as the haplotype pattern driving the significant SNPster association on MMU10 (Figure 3C). *Socs2* has been shown to promote neurogenesis and enhance neurite outgrowth, a function relevant to depression: hippocampal volume is often reduced in depressed patients [81], and antidepressants may alleviate depression by promoting neurogenesis [82].

Although we found no coding SNPs in the *Socs2* genomic sequence and no haplotype group-associated gene expression differences, it is possible that the noncoding SNPs that we identified affect multiple other functions, including protein conformation and stability, mRNA stability, promoter element binding frequency, and miRNA binding [83]. In a recent study, *Cyp2c29* was identified as the most likely candidate for inter-strain differences in warfarin metabolism by haplotype association mapping, but no strain-specific polymorphisms were identified in the coding region of the gene and no differences in gene expression were observed [84]. Therefore, non-coding SNPs are capable of affecting protein expression or function without necessarily altering mRNA expression.

Recent large-scale strain studies have shown that inbred mouse strains represent a diversity of physiological phenotypes similar to those found in human populations [21], [85]. The present results extend this observation to the behavioral realm, and suggest that inbred mouse strains will be a valuable resource for modeling the variability of human behavioral traits. Furthermore, the availability of phenotyping data for such a large number of strains can be used to inform strain selection for F2 mapping crosses, and may also be useful for identifying appropriate strains for testing behavior-modifying drugs.

## Supporting Information

Table S1 Strains phenotyped for behavioral and physiological characteristics - Average body weight and baseline corticosterone levels (µg/dl) are provided.
(0.02 MB XLS)

Table S2 Average results for TST immobility, OF center time, and OF distance traveled - Complete data are available under the file Pletcher1 in the Mouse Phenome Database.
(0.03 MB XLS)
